# Bone Morphogenetic Proteins 2/4 Are Upregulated during the Early Development of Vascular Calcification in Chronic Kidney Disease

**DOI:** 10.1155/2018/8371604

**Published:** 2018-04-12

**Authors:** Xiao Wei, Weihua Wu, Li Li, Jiaru Lin, Qi Liu, Linwang Gan, Santao Ou

**Affiliations:** Department of Nephrology, The Affiliated Hospital of Southwest Medical University, Luzhou, Sichuan 646000, China

## Abstract

Vascular calcification is a main cause of increased cardiovascular morbidity and mortality in chronic kidney disease (CKD) patients. This study aimed to investigate the role of the bone morphogenetic protein (BMP) signaling pathway in the early development of vascular calcification in CKD. A CKD vascular calcification rat model was established by providing rats with a 1.8% high-phosphorus diet and an intragastric administration of 2.5% adenine suspension. The kidney and aortic pathologies were analyzed. Blood biochemical indicators, serum BMP-2 and BMP-4 levels, and aortic calcium content were determined. The expression levels of BMP-2, BMP-4, bone morphogenetic protein receptor-IA (BMPR-IA), and matrix Gla protein (MGP) in aorta were examined by quantitative real-time polymerase chain reaction and immunohistochemistry. Compared with the normal control (Nor) rats, the CKD rats exhibited a significantly decreased body weight and an increased kidney weight as well as abnormal renal function and calcium-phosphorus metabolism. Aortic von Kossa and Alizarin red staining showed massive granular deposition and formation of calcified nodules in aorta at 8 weeks. The aortic calcium content was significantly increased, which was positively correlated with the serum BMP-2 (*r* = 0.929; *P* < 0.01) and serum BMP-4 (*r* = 0.702; *P* < 0.01) levels in CKD rats. The rat aortic BMP-2 mRNA level in the CKD rats was persistently increased, and the BMP-4 mRNA level was prominently increased at the 4th week, declining thereafter. Strong staining of BMP-2, BMP-4, BMPR-IA, and MGP proteins was observed in the tunica media of the aorta from the 4th week after model induction. In conclusion, activation of the BMP signaling pathway is involved in the early development of vascular calcification in CKD. Therefore, elevated serum BMP-2 and BMP-4 levels may serve as serum markers for CKD vascular calcification.

## 1. Introduction

The prevalence of chronic kidney disease (CKD) has increased every year and has become a global public health problem, affecting 8–16% of adults worldwide [1–3]. Cardiovascular disease remains the leading cause of mortality in CKD patients, especially those with end-stage renal disease, who have a death risk that is 20–30 times higher than that of the general population [4]. Mineral bone disorder in early CKD patients promotes vascular osteoblastic transition, increases the secretion of osteocytic proteins, and eventually stimulates the formation of vascular calcification [5]. The prevalence of vascular calcification ranges from 40% in stage 3 CKD patients to up to 80–90% in stage 5 CKD patients [6]. Furthermore, vascular calcification has been found as an independent risk factor for cardiovascular morbidity and mortality in CKD patients [7].

Vascular calcification is an active, complex biological process that is highly regulated; the central step involves the transdifferentiation of the contractile phenotype of vascular smooth muscle cells (VSMCs) in the media toward an osteoblast-like state [8, 9]. Thus, CKD-related vascular calcification is manifested mostly as arterial media calcification [10, 11]. A variety of risk factors, such as hyperphosphatemia, secondary hyperparathyroidism, chronic inflammation, and oxidative stress, may induce the formation of vascular calcification in CKD patients [12, 13] and are accompanied by the increased expression of bone matrix proteins, including bone morphogenetic proteins (BMPs), osteopontin, osteoprotegerin, and osteocalcin. During this process, various signaling pathways, including the BMPs/Smad1/5/8, Notch/Msx2, and Wnt/beta-catenin signaling pathways, and the downstream molecules Runx2 and Osterix are activated to participate in the initiation and progression of vascular calcification [14–16].

BMPs may play an important role in the pathogenesis of vascular calcification [17–20]. BMPs are members belonging to the transformation growth factor-beta superfamily. They are essential for osteogenesis and heterotopic ossification [21, 22]. BMPs bind to type II and type I serine-threonine kinase receptors (bone morphogenetic protein receptor-IA (BMPR-IA), BMPR-IB, activin receptor-like kinase-2 (ALK-2), and ALK1) to form specific complexes. The complexes regulate the phosphorylation of Smad1/5/8 and then combine with Smad4 protein, which together translocate to the nucleus, where they are involved in osteogenesis and other biological processes [22]. Both BMP-4 and BMP-2 share structure similarity and have osteogenic and ectopic bone formation activities. Matrix Gla protein (MGP), an inhibitor of BMPs, inhibits VSMC osteogenesis and calcification by antagonizing BMP-2 and BMP-4 [23]. MGP can also prevent ectopic mineralization by directly combining with hydroxyapatite crystals to reduce calcium salt crystal deposition [24]. However, the dynamic expression changes in BMPs and their receptors during the process of CKD-related vascular calcification remain largely unknown.

In this study, we established a CKD vascular calcification rat model by providing rats with a 1.8% high-phosphorus diet and an intragastric administration of 2.5% adenine suspension. The dynamic changes in the expression levels of BMP-2 and BMP-4 as well as their receptor BMPR-IA and inhibitor MGP were monitored. By exploring the molecular mechanism of vascular calcification in CKD, this study provides evidence for potential strategies that prevent cardiovascular complications in CKD.

## 2. Materials and Methods

### 2.1. Animals and Grouping

Specific pathogen-free male Sprague-Dawley rats (*n* = 55, 7-8 weeks old, 190–270 g; certificate number: SYXK 2013-065) were obtained from the animal center at Southwest Medical University (Luzhou, China). The experimental protocol was approved by the ethics committee of the Animal Care and Use Committee at Southwest Medical University. All rats were provided free access to food and water and housed under a 12/12 h light-dark cycle at 18–25°C.

### 2.2. Animal Model

The rats were kept under observation for ten days prior to the start of the experiment. They were then randomly divided into two groups, that is, normal controls (Nor group, *n* = 20) and CKD rats with vascular calcification (CKD group, *n* = 35). Adenine (Sigma Chemical Co., St. Louis, MO, USA) was dissolved in distilled water to prepare the adenine suspension. The rats in the CKD group were fed with a 1.8% high-phosphorus diet and given 2.5% adenine suspension (220 mg/kg/d) by gavage daily for 4 weeks and then every other day for the next 4 weeks. The rats in the Nor group were fed with standard chow and intragastrically given the same volume of saline at the same frequency. During the experiment, food and water intake by the rats, body weight, and general conditions of the rats were monitored.

### 2.3. Determination of 24-Hour Urinary Protein Excretion

The rats were placed in metabolic cages for 24-hour urine collection at the end of 2, 4, 6, and 8 weeks, respectively. Urine protein concentrations were determined by an Advia 2400 automatic biochemistry analyzer (Siemens, Erlangen, Germany).

### 2.4. Biochemical Analysis

At the end of 2, 4, 6, and 8 weeks, respectively, rats (Nor group, *n* = 5; CKD group, *n* = 6) were anesthetized by an intraperitoneal injection of 2% pentobarbital sodium (Sigma Chemical Co., St. Louis, MO, USA) at a dose of 30–60 mg/kg. The blood samples collected from the abdominal aorta were analyzed by an automatic biochemistry analyzer (Advia 2400, Siemens, Erlangen, Germany) for serum creatinine (Scr), blood urea nitrogen (BUN), cystatin C, serum calcium, and phosphorus. Additional blood samples were centrifuged at 3000 rpm/min and 4°C for 5 min. The supernatant was collected, labeled, and then stored at −20°C until further processing.

### 2.5. Preparation of Kidney and Aorta Samples

After blood sample collection, the kidneys were harvested and fixed in 10% neutral formalin solution for hematoxylin and eosin staining. The aorta from each rat was resected into three sections, which were preserved in 10% neutral formalin solution, RNA preservation solution at −20°C, or placed at −80°C, respectively.

### 2.6. Histological Examination

Rat kidney and aortic tissues were fixed in 10% neutral formalin solution and embedded in paraffin. Then, paraffin-embedded tissues were cut into 4 *μ*m sections, which were deparaffinized and rehydrated. The renal tissues were stained with hematoxylin and eosin to evaluate the severity of renal lesions under light microscopy (Olympus, Japan).

Aortic tissues were stained with von Kossa and Alizarin red staining for the evaluation of calcification, separately. For von Kossa staining, the sections were covered with 5% silver nitrate (Shanghai Yuanye Biotech., China) for 30 min and then exposed to ultraviolet light for 60 min. After washing, the sections were covered with 5% sodium thiosulfate and incubated for 5 min. The sections were washed again and counterstained with nuclear fast red (Solarbio, Beijing, China) to visualize the nuclei. For Alizarin red staining, after deparaffinization and washing, the tissues were stained with 2% Alizarin red solution for 30 min at room temperature with gentle rotation. The sections were mounted on glass slides, and images were observed under a light microscope (Nikon, Japan).

### 2.7. Enzyme-Linked Immunosorbent Assay (ELISA)

For the measurement of BMP-2 and BMP-4, blood samples were immediately centrifuged at 3000 rpm for 5 min, and the serum was stored at −80°C prior to analysis. The BMP-2 and BMP-4 concentrations were determined using ELISA kits (Wuhan Douceur Biotech, China), according to the manufacturer's instructions. The optical density of each well was determined by using a microplate reader (Bio-Rad Laboratories, Hercules, CA, USA) at 450 nm. Calculation of serum BMP-2 and BMP-4 concentrations was performed by reference to standard curves constructed with the BMP-2 and BMP-4 standards provided in the kits.

### 2.8. Determination of Calcium Content in Aorta Samples

Portions of aorta were excised to prepare aortic homogenate, and then the supernatants were extracted. The calcium content in the supernatant was determined by using a calcium determination kit (Nanjing Jiancheng Biotech., China) and atomic absorption spectrophotometry at a wavelength of 610 nm. The aortic calcium content was expressed as mmol/g of protein (mmol/gprot).

### 2.9. Quantitative Real-Time Polymerase Chain Reaction (qRT-PCR)

Total RNA was extracted from frozen aortic tissues (50 mg) using TRIzol reagent and was reverse-transcribed into complementary DNA (cDNA) using the RevertAid First Strand cDNA Synthesis Kit (Thermo Scientific, Rockford, IL, USA). The primer sequences used for RT-PCR analyses are shown in Table 1. DNA amplification was performed on a StepOne Real-Time PCR System (Eppendorf, Germany) with a SYBR Premix Ex Taq II kit (Tli RNaseH Plus, Takara, Otsu, Japan) using the following thermal conditions: an initial step at 95°C for 10 min, followed by 40 cycles of 95°C for 15 s, 55°C for 15 s, and 72°C for 30 s. The relative amount of mRNA in each sample was calculated using the 2^−ΔΔCT^ method and was corrected by reference to the expression of GAPDH (loading control).

### 2.10. Immunohistochemical Analysis

The following primary antibodies were used: rabbit anti-rat polyclonal antibody BMP-2 and BMP-4 (Abcam, Cambridge, MA, USA); rabbit anti-rat polyclonal antibody BMPR-IA (Sigma Chemical Co., St. Louis, MO, USA); and rabbit anti-rat polyclonal antibody MGP (Abcam, Cambridge, MA, USA).

The paraffin-embedded aortic tissues were cut into 4 *μ*m thick sections and then deparaffinized in xylene and dehydrated in a graded series of ethanol solutions. After antigen retrieval, the sections were incubated with primary antibody at 4°C overnight. After rinsing with phosphate-buffered saline (PBS), the sections were incubated with biotinylated secondary antibodies for 20–30 min, washed again, and covered with horseradish peroxidase-conjugated streptavidin. The reaction was visualized using 3,3′-diaminobenzidine staining. The sections were rinsed with water and counterstained with Mayer's hematoxylin. As a negative control, staining was performed in parallel using PBS instead of the primary antibody.

### 2.11. Statistical Analyses

The SPSS for Windows version 24.0 software package (SPSS Inc., USA) was used for statistical data analysis. Quantitative data are expressed as means ± standard deviations. Comparison between two groups was performed using the independent-samples *t*-test. Data were analyzed using one-way analysis of variance to evaluate intergroup differences. Correlations between data were analyzed using Pearson's test. *P* < 0.05 and *P* < 0.01 were considered to be statistically significant and highly statistically significant, respectively.

## 3. Results

### 3.1. General Conditions

The rats in the CKD group exhibited polydipsia, anorexia, polyuria, weight loss, reduced activities, and dull fur from the 2nd week; a total of nine rats died from the 2nd to 8th week (a total of 26 rats survived). In contrast, all rats in the Nor group had normal appearance and activities, and no rats died during the experimental period (*n* = 20).

### 3.2. Body and Kidney Weights of Rats

**Figure 1:**
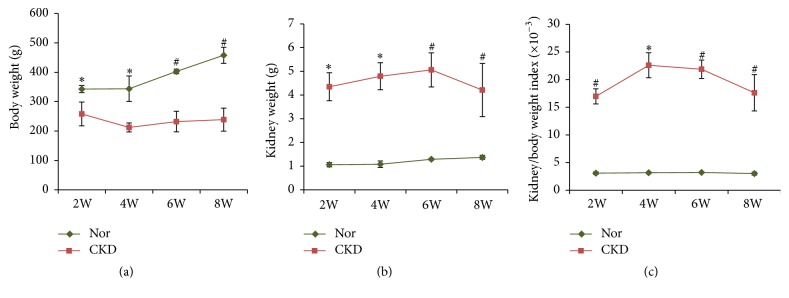
*Changes in the body weight and kidney weight.* Chronic kidney disease (CKD) rats experienced weight loss after model induction, and the body weight decreased to the lowest level at the 4th week (a). On the contrary, these rats exhibited a significantly increased kidney weight (b) and kidney/body weight index (c). ^*∗*^*P* < 0.05 and ^#^*P* < 0.01 versus the normal control (Nor) group at the same time point.

Compared with the Nor group, the body weights of the rats in the CKD group were significantly decreased at all time points (all *P* < 0.05 or *P* < 0.01; Figure 1, Supplementary Table  1), especially at the 4th week when the body weight decreased most prominently. In contrast, the kidney weights were significantly increased in the CKD group (all *P* < 0.05 or *P* < 0.01), which led to a remarkable increase in the kidney/body weight index, compared with the Nor group (all *P* < 0.05 or *P* < 0.01).

### 3.3. 24-Hour Urine Protein Excretion and Renal Function

**Figure 2:**
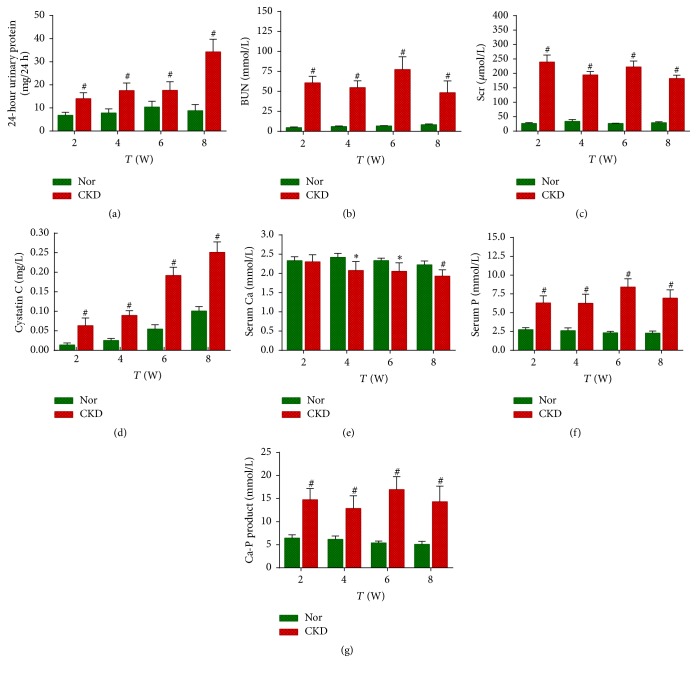
*24-hour urine protein excretion and renal function.* Rats in the chronic kidney disease (CKD) group exhibited a vigorous increase in 24-hour urine protein excretion (a), blood urine nitrogen (b), serum creatinine (c), and cystatin C (d) levels compared to those of the normal control (Nor) group. Compared with the Nor group, the blood phosphorus levels of CKD rats were significantly increased (e), accompanied by decreased blood calcium levels from the 4th week (f). These changes led to an increased calcium-phosphorus product (g). ^*∗*^*P* < 0.05 and ^#^*P* < 0.01 versus the Nor group at the same time point. BUN, blood urine nitrogen; Ca, calcium; P, phosphorus; SCr, serum creatinine.

Rats in the CKD group exhibited a vigorously increasing trend of 24-hour urine protein excretion over time after model induction, from 14.0 ± 2.61 mg/24 h at the 2nd week to 34.33 ± 5.43 mg/24 h at the 8th week; these values were significantly higher than those of the Nor group (*P* < 0.01 at all time points; Figure 2(a), Supplementary Table  2).

Similarly, rats in the CKD group had significantly higher levels of BUN, Scr, and cystatin C than those of the Nor group (all *P* < 0.01 at all time points; Figures 2(b)–2(d), Supplementary Table 2), indicating impaired renal function of the rats in the CKD group.

Compared with the control group, the blood phosphorus level of rats in the CKD group was significantly increased (*P* < 0.01 at all time points), whereas the blood calcium level was significantly decreased from the 4th week (*P* < 0.05 at the 4th and 6th weeks and *P* < 0.01 at the 8th week). These changes led to an increased calcium-phosphorus product level (*P* < 0.01 at all time points; Figures 2(e)–2(g), Supplementary Table 2).

### 3.4. Renal Pathology

**Figure 3:**
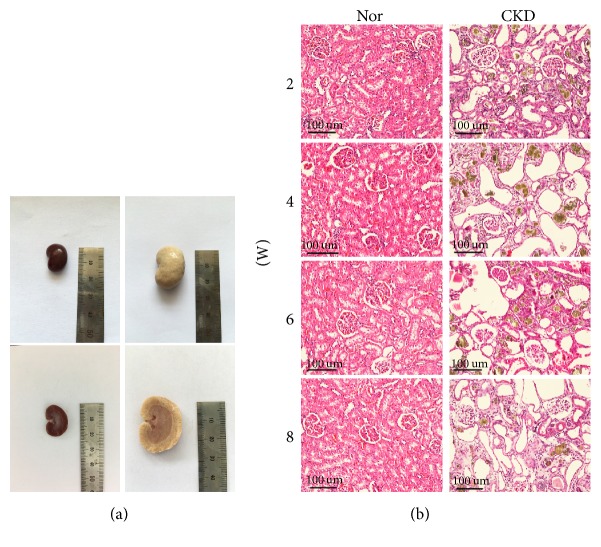
*Renal pathology was examined by gross observation and light microscopy.* (a) By observing gross specimens, the kidneys in the chronic kidney disease (CKD) group were significantly enlarged, with a grey pale color, rough surface, lack of elasticity, and hard texture. (b) Under light microscopy, dilated tubules were visible in the CKD group from the end of the 2nd week. At the end of the 4th week, aggravated tubular dilation and accumulated brownish or yellowish granular deposits were observed, accompanied by mild interstitial fibrosis and partial glomerular atrophy. With disease progression over time, the histological abnormalities included structural disorder, diffuse tubular dilation, excessive accumulation of granular deposits, tubular epithelial degeneration, necrosis, significant interstitial fibrosis, infiltration of inflammatory cells, severe glomerular atrophy, and a dilated lumen at the end of the 8th week.

By observation of the gross specimen, the kidneys in the Nor group had a normal size, dark red color, bright and smooth surface, and soft texture. In contrast, the kidneys in the CKD group were significantly enlarged, with a grey pale color, rough surface, lack of elasticity, and hard texture (Figure 3(a)).

Under light microscopy, the normal kidneys had an intact structure, normal appearance of renal nephrons, and clear cortex-medulla boundaries (Figure 3(b)). But in the CKD group, dilated tubules were visible from the end of the 2nd week, predominantly in the proximal renal tubules, accompanied by brownish or yellowish granular deposits. An early glomerular change was not obvious. At the end of the 4th week, aggravated tubular dilation and accumulated brownish or yellowish granular deposits were observed, accompanied by mild interstitial fibrosis and partial glomerular atrophy. With disease progression over time, the histological abnormalities included structural disorder, diffuse tubular dilation, excessive accumulation of granular deposits, tubular epithelial degeneration, necrosis, significant interstitial fibrosis, infiltration of inflammatory cells, severe glomerular atrophy, and a dilated lumen at the end of the 8th week.

### 3.5. Aortic Pathology

**Figure 4:**
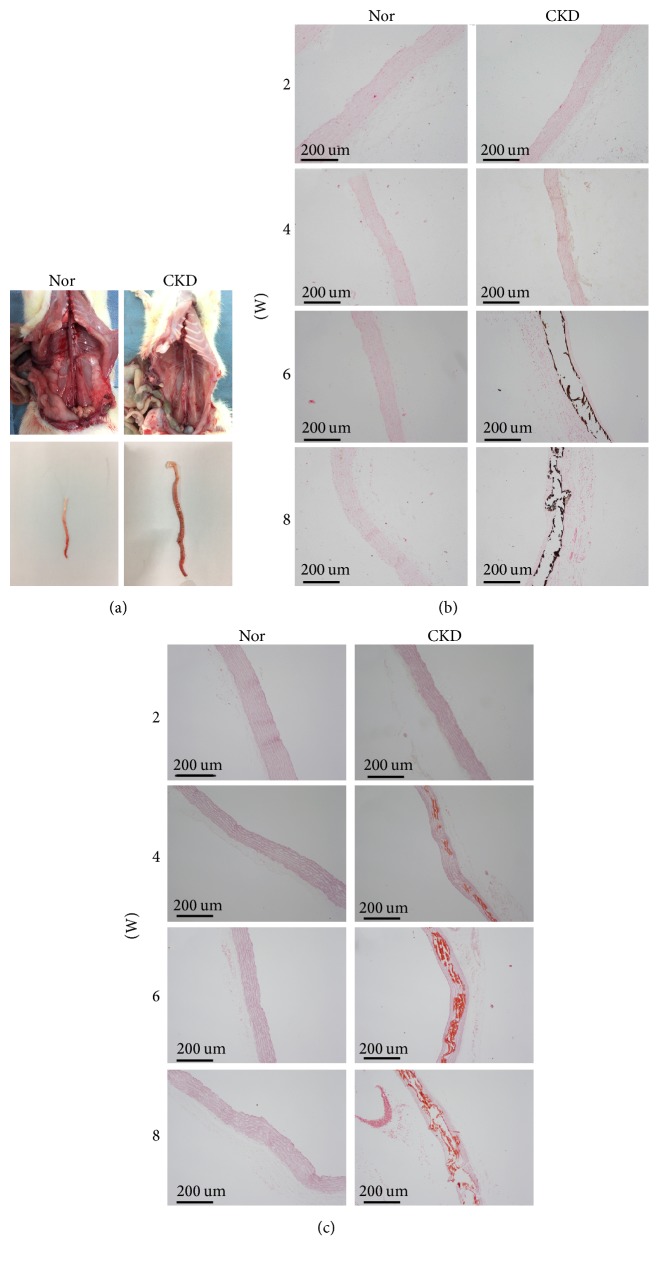
*Aortic pathology was examined by gross observation, aortic von Kossa staining, and aortic Alizarin red staining.* (a) Observation of gross aortic specimens revealed aortic aneurysm-like changes in the aorta in the chronic kidney disease (CKD) group; (b) aortic von Kossa staining showed gradually increased black granular deposition and formation of calcified nodules in the CKD group; (c) aortic Alizarin red staining showed progressively increased orange-colored granular deposition and formation of calcified nodules in the CKD group.

Observation of the gross aortic specimen revealed that the aorta was smooth, with a good elasticity and a normal appearance. However, the aorta in the CKD group from the 4th week became rugged or crooked, swollen, and inflexible. At the 6th week, the aorta revealed aortic aneurysm-like changes, with a reduced refractive index, poor elasticity, rough surface, increased vessel brittleness, and formation of calcified nodules (Figure 4(a)).

The results of aortic von Kossa staining showed no deposits in the aorta of the Nor rats (Figure 4(b)). In the CKD group, the vascular morphology was basically normal at the 2nd week, and no black deposits were observed. At the 4th week, a small amount of black granular deposits was dispersed in the aorta. The aortic lesions became more severe over time, with increased vessel stiffness, ruptured smooth muscle fibers, massive black granular deposition, and formation of calcified nodules observed at the 8th week.

Similarly, the aortic Alizarin red staining results showed no deposits in the aorta of the Nor rats (Figure 4(c)). In the CKD group, a small amount of orange-colored deposits was observed from the 4th week, which progressively increased over time. At the end of the 8th week, massive orange-colored granular deposition and formation of calcified nodules were observed.

### 3.6. Serum BMP-2 and BMP-4 Levels by ELISA

**Figure 5:**
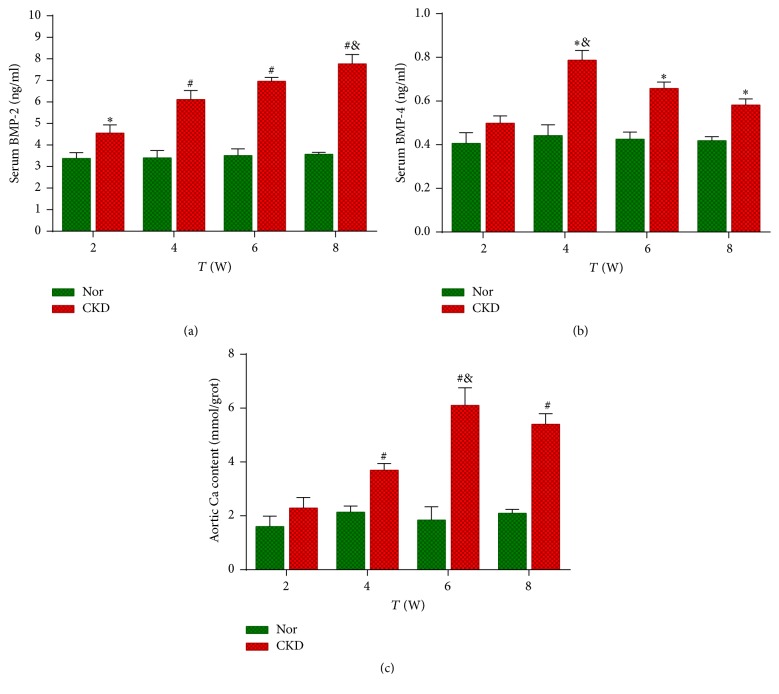
*Serum BMP-2 and BMP-4 levels by ELISA and aortic calcium content.* (a) Serum BMP-2 concentration; (b) serum BMP-4 concentration; (c) aortic calcium content. ^*∗*^*P* < 0.05 and ^#^*P* < 0.01 versus the normal control (Nor) group at the same time point. ^&^Intergroup comparison was made: for BMP-2, *P* < 0.01 versus 2 and 4 weeks and *P* < 0.05 versus 6 weeks; for BMP-4, *P* < 0.05 versus 2, 6, and 8 weeks; for aortic calcium content, *P* < 0.05 versus 2 and 4 weeks.

There were no significant changes in the serum BMP-2 levels in the Nor group during the experimental period (*P* > 0.05, Figure 5(a)). The serum BMP-2 level in the CKD group was significantly increased at the 2nd week, compared with that of the Nor group (*P* < 0.05). Then, the serum BMP-2 concentration in the CKD rats was persistently increased until the 8th week (*P* < 0.01 as compared with the Nor group at 4, 6, and 8 weeks; Supplementary Table 3).

The serum BMP-4 level was slightly increased from the 2nd week in the CKD rats, although it failed to achieve statistical significance (*P* > 0.05, Figure 5(b)), and then it reached its peak level at the 4th week and gradually declined thereafter. Nonetheless, the BMP-4 levels in the CKD group were significantly higher than those of the Nor group at all time points except for the 2nd week (*P* < 0.05).

### 3.7. The Aortic Calcium Content and Its Correlation with the BMP-2 and BMP-4 Levels

Compared with the Nor group, the aortic calcium content of the CKD group was significantly increased from the 4th week (*P* < 0.01). As the disease progressed, the aortic calcium content in the CKD rats continually increased to the 6th week (*P* < 0.01 versus the Nor group) and then decreased to the 8th week (*P* < 0.01 versus the Nor group; Figure 5(c), Supplementary Table 3).

Pearson's correlation analysis revealed positive correlations between the aortic calcium content and the serum BMP-2 (*r* = 0.929; *P* < 0.01) and serum BMP-4 (*r* = 0.702; *P* < 0.01) levels in the CKD rats.

### 3.8. BMP-2 and BMP-4 mRNA Levels in Aorta Detected by qRT-PCR

**Figure 6:**
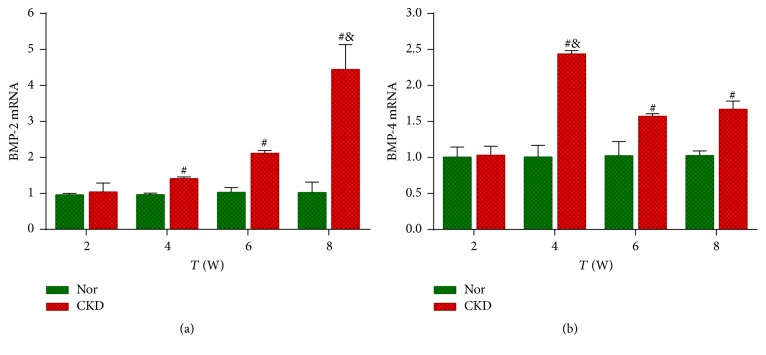
*The mRNA levels of BMP-2 (a) and BMP-4 (b) in the aorta by quantitative real-time PCR analysis.* ^#^ *P* < 0.01 versus the normal control (Nor) group at the same time point. ^&^Intergroup comparison was made: for BMP-2, *P* < 0.01 versus 2, 4, and 6 weeks; for BMP-4, *P* < 0.01 versus 2, 6, and 8 weeks.

The mRNA levels of BMP-2 and BMP-4 were stably expressed in the normal aorta during the whole experimental period. However, the rat aortic BMP-2 mRNA level began to increase at the 4th week (*P* < 0.01 versus the Nor group) in the CKD rats and persistently increased thereafter (*P* < 0.01 versus the Nor group at the 6th and 8th weeks; Figure 6(a)). Meanwhile, the rat aortic BMP-4 mRNA level increased prominently at the 4th week in the CKD rats (*P* < 0.01 versus the Nor group) and declined thereafter, although it was still higher than that of the Nor group (*P* < 0.01 at the 6th and 8th weeks; Figure 6(b)).

### 3.9. BMP-2, BMP-4, BMPR-IA, and MGP Protein Levels in Aorta Detected by Immunohistochemistry

**Figure 7:**
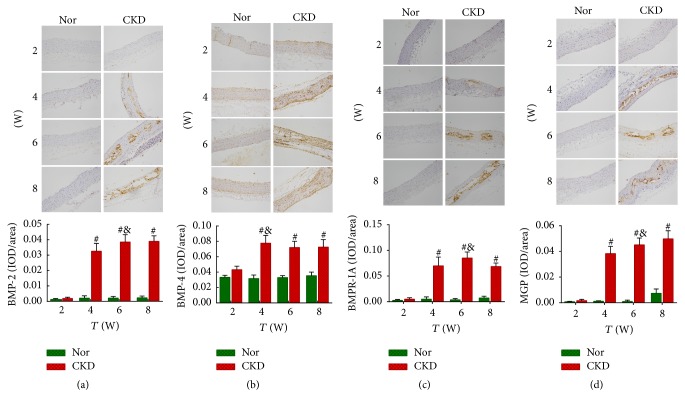
*The BMP-2, BMP-4, BMPR-IA, and MGP expression levels in the aorta by immunohistochemical analysis.* BMP-2 (a), BMPR-IA (b), and MGP (c) proteins were barely expressed, and BMP-4 (d) was weakly expressed in the normal aorta. Strong staining of BMP-2, BMP-4, BMPR-IA, and MGP proteins was observed in the vascular smooth muscle layer (tunica media) of the aorta at 4, 6, and 8 weeks after chronic kidney disease (CKD) model induction. Magnification, ×200. ^#^*P* < 0.01 versus the normal control (Nor) group at the same time point. ^&^Intergroup comparison was made: for BMP-2, BMPR-IA, and MGP, *P* < 0.01 versus 2 weeks and *P* < 0.05 versus 4 weeks; for BMP-4, *P* < 0.01 versus 2 weeks.

BMP-2, BMPR-IA, and MGP proteins were barely expressed and BMP-4 was weakly expressed in the normal aorta (Figure 7). Strong staining of BMP-2, BMP-4, BMPR-IA, and MGP proteins was observed in the vascular smooth muscle layer (tunica media) of the aorta at the 4th, 6th, and 8th weeks, suggesting significantly increased expression of these proteins from the 4th week after model induction.

## 4. Discussion

CKD is a progressive loss of kidney function over months or years and is pathologically characterized by glomerular sclerosis, tubular and interstitial fibrosis, and inflammatory cell infiltration [25]. Thus, establishment of a reliable CKD model is necessary to facilitate understanding of the disease evolution and identification of an intervention target as well as to provide a feasible strategy for treatment. In this study, we successfully established a CKD vascular calcification rat model by providing rats with a high-phosphorus diet and an intragastric administration of adenine suspension, manifesting as impaired renal function and the presence of aortic calcification. Both serum BMP-2 and BMP-4 levels were positively correlated with the aortic calcium content. In addition, both BMP-2 and BMP-4 as well as their receptor BMPR-IA and inhibitor MGP were upregulated in the aortic lesions of CKD rats, as compared with the controls, indicating involvement of the BMP signaling pathway in CKD-related vascular calcification.

Several methods have been used to develop a CKD animal model, such as intragastric administration or feeding with adenine, 5/6 nephrectomy, and unilateral urethral obstruction [26]. Despite the diversity of methods, they have their own features and are selectively used according to different study aims. The adenine-induced CKD animal model is highly reproducible; in particular, it mimics, but in an accelerated manner, the progression of chronic renal failure and produces metabolic abnormalities. Adenine is converted by xanthine oxidase into 2,8-dihydroxyadenine after intrahepatic metabolism, which is insoluble in water. The metabolites may deposit within the renal tubules and expand the tubule lumen, thus causing renal insufficiency [27]. In this study, the rats were intragastrically administered with 2.5% adenine suspension daily for 4 weeks and then every other day for the next 4 weeks. The rat models developed CKD within a short time period, with a high success rate and a relatively low mortality. The CKD rats at the early stage exhibited enlarged appearance of the kidneys, with a grey pale color, rough surface, and hard texture, which were similar to the characteristics of obstructive nephropathy. The renal lesions worsened with persistent damage from adenine administration, as evidenced by glomerular sclerosis or atrophy, excessive accumulation of granular deposits, interstitial fibrosis, inflammatory cell infiltration, and reduction of vessels at the late stage. Consistently, the CKD rats had increased levels of 24-hour urine protein excretion and abnormal biochemical renal indicators (Scr, BUN, and cystatin C). These data revealed successful establishment of the CKD rat model.

High phosphorus content that always accompanies CKD has been reported as a direct contributor to vascular calcification [28–30]. Hyperphosphatemia may promote intracellular phosphorus influx, dependent on or independent of type III sodium-dependent phosphate cotransporter-1, leading to a phenotypic transformation of VSMCs into osteogenic-like cells and subsequent formation of vascular calcification [31]. Yamada et al. [32] have explored the malnutrition and inflammation states as well as vascular calcification in adenine-induced CKD rats fed diets with high phosphorus concentrations. In addition, Liu et al. [33] have successfully established a CKD rat model of vascular calcification through the intragastric administration of 250 mg/kg adenine suspension and feeding with 1.8% high-phosphorus forage. In this study, we observed the presence of aortic calcification after feeding the rats with a high-phosphorus diet. From the 4th week, the aorta underwent an aortic aneurysm-like change in appearance by gross inspection, with reduced elasticity and rough surface. The granular deposition with calcified nodules was verified by von Kossa and Alizarin red staining in the aortic lesions, which was progressively exaggerated over time. Consistently, the aortic calcium content increased in the rat models. These data suggest the presence of aortic calcification.

Activation of the vascular BMP signaling pathway has been recognized as an important mechanism of vascular calcification in CKD [17–19]. Increased expression levels of BMP-2 and BMP-4 as well as their receptors (ALK-1, 2) and inhibitor (MGP) have been observed on the endothelial side of the aorta in diabetic rats, accompanied by increased osteogenesis and aortic calcium accumulation, suggestive of a link between vascular calcification and BMP activation [14]. Inhibition of BMP signaling by a BMP antagonist (LDN-193189 or ALK3-Fc) has led to reduced vascular calcification and improved survival in MGP(−/−) mice, revealing a critical role of the BMP signaling pathway in the development of vascular calcification [23]. Our previous study provided evidence supporting involvement of the BMP-2/Smad1/Runx2/Osterix signaling pathway in the development of vascular calcification in diabetic nephropathy rats [34]. In this study, the serum BMP-2 and BMP-4 levels of CKD rats were significantly increased, compared with the controls. Moreover, elevated serum BMP-2 and BMP-4 expression levels were positively correlated with the aortic calcium content (*r* = 0.929 and *P* < 0.01; *r* = 0.702 and *P* < 0.01, resp.). Our findings were consistent with the results of Stahls et al. who found a significantly increased serum BMP-4 level that positively correlated with the coronary artery calcium scores in patients with CKD and coronary artery calcification.

Despite uncertainty, induction of inflammation, excessive oxidative stress, and apoptosis are currently considered as main mechanisms by which BMPs are involved in vascular calcification. With elevated expression of BMP-4 in endothelial cells, a complex series of events occurred, including NADPH oxidase-dependent reactive oxygen species (ROS) generation and subsequent activation of nuclear factor-kappa B and intercellular adhesion molecule 1, consequently causing the accumulation of leukocytes and the critical process of early atherosclerosis [35, 36]. In addition, BMP-4 might induce oxidative stress and apoptosis of endothelial cells through activation of caspase-3 in a ROS/p38MAPK/JNK-dependent signaling cascade, leading to vascular calcification [20]. In this study, the immunohistochemical results showed that BMP-2 protein was barely expressed in the normal aorta but strongly expressed in the tunica media of the aorta in CKD rats, whereas BMP-4 was weakly expressed in the normal aorta, and positive staining of BMP-4 protein was observed in almost the whole wall of the aorta in CKD rats. Interestingly, in the CKD rats, the aortic BMP-2 mRNA level began to increase at the 4th week and persistently increased thereafter, whereas the BMP-4 mRNA level increased prominently at the 4th week and slightly declined thereafter, suggesting inconsistent trends of changes in BMP-2 and BMP-4. With time, we also noticed an increasingly enhanced expression of MGP, an inhibitor of BMP-2 and BMP-4, probably due to a compensatory mechanism of the body. This might also explain the phenomenon that BMP-4 upregulation occurred much earlier than BMP-2 did, but its level decreased after a peak expression at the 4th week, perhaps due to a weaker osteogenic activity or stronger binding to MGP, resulting in more obvious BMP-4 inhibition. However, the exact mechanism underlying the balance between BMPs and their inhibitors in the formation of vascular calcification needs further investigation.

This study has some limitations. First, there was a lack of* in vitro* experiments to verify the molecular process of the BMP signaling pathway during calcification, especially the downstream targets. Second, no evidence was provided regarding the possible difference in osteogenesis activity of BMP-2 and BMP-4 as well as their link with MGP when involved in CKD-related vascular calcification. Finally, previous studies have shown that BMP-2 and BMP-4 are involved in the occurrence of vascular calcification [15, 37]. In contrast, other studies have shown that inhibition of the BMP-2 and/or BMP-4 signal transduction pathways can indeed prevent the medial vascular calcification [23, 38, 39]. Thus, silencing of the BMP signaling pathway by antagonists or genetic manipulation might be helpful to elucidate the changes in aortic calcification. However, we did not validate this hypothesis, which will be conducted in the future.

In conclusion, activation of the BMP signaling pathway was involved in the early development of vascular calcification in CKD rats. The elevated serum BMP-2 and BMP-4 levels were positively correlated with the aortic calcium content. Thus, they may serve as serum markers for aortic calcification in CKD. Further efforts are needed to identify the specific targets in the BMP signaling pathway for developing an effective therapeutic strategy against aortic calcification in CKD by direct blockade of this pathway.

## Figures and Tables

**Table 1:** The primer sequences used for polymerase chain reaction analysis.

Gene	Primer sequences	Size
BMP-2		
Forward	5′-GATGAGTTTCTCACATCTGCGGAG-3′	155 bp
Reverse	5′-GTGTCCAATAGTCTGGTCACAGGA-3′	
BMP-4		
Forward	5′-CTGTGAGGAGTTTCCATCACGAAG-3′	124 bp
Reverse	5′-TCTGCAGAGGAGATCACCTCATTC-3′	
GAPDH		
Forward	5′-GGTGAAGGTCGGTGTGAACG-3′	233 bp
Reverse	5′-CTCGCTCCTGGAAGATGGTG-3′	

## Supplementary Materials

Supplementary Materials Supplementary Table  1: body and kidney weights of rats. Supplementary Table  2: 24-hour urine protein excretion, renal function, and calcium-phosphate metabolism. ^*∗*^*P* < 0.05 and ^#^*P* < 0.01 versus the Nor group at the same time point. Supplementary Table  3: bone morphogenetic proteins 2/4 levels and aortic calcium content
