# Supplementary material for: Bone Morphogenetic Proteins 2/4 Are Upregulated during the Early Development of Vascular Calcification in Chronic Kidney Disease
Source: Biomed Res Int. 2018 Apr 12;2018:8371604. doi: 10.1155/2018/8371604 (PMC5925148; doi:10.1155/2018/8371604)
Supplement: Supplementary Materials — Supplementary Table 1: body and kidney weights of rats. Supplementary Table 2: 24-hour urine protein excretion, renal function, and calcium-phosphate metabolism. ∗P < 0.05 and #P < 0.01 versus the Nor group at the same time point. Supplementary Table 3: bone morphogenetic proteins 2/4 levels and aortic calcium content [file 8371604.f1.docx]

**Supplementary Table 1** Body and kidney weights of rats

| Group | N | Week | Body weight  (g) | Kidney weight  (g) | Kidney/body weight index  (×10^-3^) |
| --- | --- | --- | --- | --- | --- |
| Nor | 5 | 2^nd^ | 342.70±12.17 | 1.06 ± 0.99 | 3.08 ± 0.23 |
|  | 5 | 4^th^ | 343.88 ± 43.37 | 1.08 ± 0.14 | 3.16 ± 0.17 |
|  | 5 | 6^th^ | 402.58 ± 7.70 | 1.29 ± 0.04 | 3.20 ± 0.12 |
|  | 5 | 8^th^ | 457.76 ± 27.34 | 1.37 ± 0.08 | 3.01 ± 0.28 |
| CKD | 6 | 2^nd^ | 257.97 ± 40.64^*^ | 4.35 ± 0.59^*^ | 16.97 ± 1.36^#^ |
|  | 6 | 4^th^ | 212.15 ± 15.32^*^ | 4.79 ± 0.57^*^ | 22.58 ± 2.26^#^ |
|  | 6 | 6^th^ | 232.08 ± 35.05^#^ | 5.06 ± 0.72^#^ | 21.87 ± 1.66^#^ |
|  | 6 | 8^th^ | 238.64 ± 39.15^#^ | 4.21 ± 1.12^#^ | 17.61 ± 3.27^#^ |

^*^P < 0.05, ^#^P < 0.01 vs. the normal control (Nor) group at the same time point.

**Supplementary Table 2** 24-h urine protein excretion, renal function, and calcium-phosphate metabolism

|  | Normal control (Nor) group | | | |  | Chronic kidney disease (CKD) group | | | |
| --- | --- | --- | --- | --- | --- | --- | --- | --- | --- |
|  | 2 weeks  (n = 5) | 4 weeks  (n = 5) | 6 weeks  (n = 5) | 8 weeks  (n = 5) |  | 2 weeks  (n = 6) | 4 weeks  (n = 6) | 6 weeks  (n = 6) | 8 weeks  (n = 6) |
| 24-h urine protein  excretion, mg/24 h | 6.40 ± 1.14 | 7.80 ± 1.79 | 9.40 ± 1.34 | 8.8 ± 2.68 |  | 14.0 ± 2.61^#^ | 17.50 ± 3.33^#^ | 17.67 ± 3.67^#^ | 34.33 ± 5.43^#^ |
| BUN, mmol/L | 4.73 ± 0.85 | 6.12 ± 0.75 | 6.83 ± 0.37 | 8.34 ± 0.94 |  | 60.78 ± 8.18^#^ | 54.77 ± 8.43^#^ | 77.34 ± 15.94^#^ | 48.41 ± 14.66^#^ |
| SCr, umol/L | 26.67 ± 3.0 | 33.72 ± 6.29 | 26.42 ± 1.18 | 29.26 ± 3.35 |  | 239.54 ± 24.40^#^ | 194.74 ± 11.70^#^ | 222.62 ± 20.33^#^ | 182.58 ± 11.10^#^ |
| Cystatin-C, mg/L | 0.014 ± 0.053 | 0.025 ± 0.005 | 0.055 ± 0.011 | 0.101 ± 0.011 |  | 0.063 ± 0.020^#^ | 0.090 ± 0.012^#^ | 0.192 ± 0.021^#^ | 0.251 ± 0.027^#^ |
| Serum Ca, mmol/L | 2.33 ± 0.10 | 2.42 ± 0.10 | 2.33 ± 0.62 | 2.22 ± 0.10 |  | 2.30 ± 0.18 | 2.08 ± 0.23^*^ | 2.06 ± 0.22^*^ | 1.93 ± 0.16^#^ |
| Serum P, mmol/L | 2.76 ± 0.25 | 2.56 ± 0.28 | 2.32 ± 0.17 | 2.30 ± 0.28 |  | 6.43 ± 1.01^#^ | 6.23 ± 1.20^#^ | 8.26 ± 1.02^#^ | 7.39 ± 1.28^#^ |
| Calcium-phosphorus  product, mmol/L | 6.45 ± 0.71 | 6.18 ± 0.73 | 5.41 ± 0.40 | 5.10 ± 0.64 |  | 14.77 ± 2.40^#^ | 12.87 ± 2.74^#^ | 16.98 ± 2.80^#^ | 14.33 ± 3.38^#^ |

BUN, blood urine nitrogen; Ca, calcium; P, phosphorus; SCr, serum creatinine.

^*^P < 0.05, ^#^P < 0.01 vs. the Nor group at the same time point.

**Supplementary Table 3** The bone morphogenetic protein (BMP)-2 and BMP-4 levels and aortic calcium content

|  | Normal control (Nor) group | | | |  | Chronic kidney disease (CKD) group | | | |
| --- | --- | --- | --- | --- | --- | --- | --- | --- | --- |
|  | 2 weeks  (n = 5) | 4 weeks  (n = 5) | 6 weeks  (n = 5) | 8 weeks  (n = 5) |  | 2 weeks  (n = 6) | 4 weeks  (n = 6) | 6 weeks  (n = 6) | 8 weeks  (n = 6) |
| **ELISA** |  |  |  |  |  |  |  |  |  |
| Serum BMP-2, ng/mL | 3.37 ± 0.27 | 3.41 ± 0.34 | 3.51 ± 0.31 | 3.57 ± 0.10 |  | 4.56 ± 0.38* | 6.11 ± 0.42^#^ | 6.96 ± 0.18^#^ | 7.77 ± 0.44^#1^ |
| Serum BMP-4, pg/mL | 0.41 ± 0.05 | 0.44 ± 0.05 | 0.43 ± 0.03 | 0.42 ± 0.02 |  | 0.50 ± 0.03 | 0.79 ± 0.05^*2^ | 0.66 ± 0.03^*^ | 0.58 ± 0.03^*^ |
| **Spectrophotometry** |  |  |  |  |  |  |  |  |  |
| Aortic Ca content, mmol/gprot | 1.603 ± 0.387 | 2.134 ± 0.229 | 1.846 ± 0.493 | 2.099 ± 0.146 |  | 2.293 ± 0.389 | 3.699 ± 0.247^#^ | 6.105 ± 0.653^#3^ | 5.406 ± 0.389^#^ |
| **qRT-PCR** |  |  |  |  |  |  |  |  |  |
| BMP-2 mRNA | 0.962 ± 0.043 | 0.967 ± 0.040 | 1.033 ± 0.130 | 1.026 ± 0.287 |  | 1.044 ± 0.243 | 1.412 ± 0.048^#^ | 2.120 ± 0.073^#^ | 4.446 ± 0.689^#4^ |
| BMP-4 mRNA | 1.005 ± 0.139 | 1.008 ± 0.160 | 1.027 ± 0.196 | 1.029 ± 0.063 |  | 1.034 ± 0.121 | 2.442 ± 0.435^#5^ | 1.573 ± 0.035^#^ | 1.670 ± 0.114^#^ |

Ca, calcium; ^*^P < 0.05, ^#^P < 0.01 vs. the Nor group at the same time point.

^1^Inter-group comparison: P < 0.01 vs. 2 and 4 weeks; P < 0.05 vs. 6 weeks.

^2^Inter-group comparison: P < 0.05 vs. 2, 6, and 8 weeks;

^3^Inter-group comparison: P < 0.05 vs. 2 and 4 weeks;

^4^Inter-group comparison: P < 0.01 vs. 2, 4, and 6 weeks;

^5^Inter-group comparison: P < 0.01 vs. 2, 6, and 8 weeks.
